# Diet has minimal effect on *Salmonella* Typhimurium infection in the gut of a cockroach vector despite altering the microbiome

**DOI:** 10.1128/aem.01570-25

**Published:** 2025-11-26

**Authors:** Kylene Guse, Taylor Rose, Jose E. Pietri

**Affiliations:** 1 Division of Basic and Translational Sciences, Sanford School of Medicine, University of South Dakota 8191, Vermillion, South Dakota, USA; 2 Department of Entomology, Center for Urban and Industrial Pest Management, Purdue University 167064 , West Lafayette, Indiana, USA; 3 Purdue University, Institute of Inflammation, Immunology and Infectious Disease 311308 , West Lafayette, Indiana, USA; 4 Department of Biological Sciences, Purdue University 124080, West Lafayette, Indiana, USA; Norwegian University of Life Sciences, Ås, Norway

**Keywords:** cockroach, *Salmonella*, vector, pathogen, infection, diet

## Abstract

The German cockroach, *Blattella germanica*, can harbor and transmit enteric human pathogens, including *Salmonella enterica* serovar Typhimurium. German cockroaches are omnivores that subsist on highly varied diets in the field, in contrast to most arthropod vectors. Diet plays an important role in shaping the gut microenvironment across a range of animals, which can in turn affect numerous aspects of physiology, including the ability to resist infection. Yet, the impact of diet on the ability of cockroaches to maintain and transmit pathogens had not been investigated previously. Here, we tested the hypothesis that dietary differences among otherwise identical populations of *B. germanica* could lead to differences in vector competence for *S*. Typhimurium. Cockroaches were maintained on three defined formulated diets with distinct macronutrient profiles for 10 days. Food consumption was monitored during this period, and the gut microbiome was profiled by 16S rRNA amplicon sequencing. The cockroaches were then orally infected with *S*. Typhimurium, and pathogen loads in the gut and excreta were quantified. Cockroaches equally consumed formulated high-protein, high-fat, and high-carbohydrate diets in no-choice assays. Furthermore, as expected, some significant differences in microbiome composition and diversity were observed between groups of cockroaches maintained on different diets. However, despite the effects on the microbiome, no significant diet-dependent differences in the load of *S*. Typhimurium maintained in the gut or shed in the excreta were observed. Although the results provide evidence that the dietary macronutrient profile is not a major contributor to vector competence, the possibility that other natural diets could influence pathogen infection and transmission dynamics is not ruled out by this study.

**IMPORTANCE:**

German cockroaches are one of the most common structural pests worldwide, while *Salmonella enterica* serovar Typhimurium is an emerging human pathogen accounting for a significant portion of the global burden of enteric disease. Understanding the factors that contribute to the ability of cockroaches to transmit pathogens is important for infection prevention, but these remain almost entirely unknown. Here, we provide new insight into the variables involved in the vector competence of cockroaches.

## INTRODUCTION

Cockroaches, including the German cockroach, *Blattella germanica*, are associated with a number of bacteria that cause enteric disease in humans (1, 2). Among them, *Salmonella enterica* serovar Typhimurium has been shown to actively colonize the cockroach gut through a complex interplay between bacterial virulence factors, host genetics, and the microbiota, leading to sustained pathogen dissemination in the excreta (3–7).

Unlike other insect vectors, most of which have highly restrictive diets (e.g., blood and nectar), the omnivorous diet of German cockroaches can be highly variable. In the laboratory, *B. germanica* will self-select diets with a 1:2 or 1:3 ratio of protein to carbohydrates (8) and will balance nutrient imbalances within diets through selection of complementary foods when possible (9). Although it is widely thought that German cockroaches will readily consume almost any food sources available in their environment, few studies have investigated their natural diets in the field. However, nymphs in the field appear to consume higher levels of fat and lower levels of protein and carbohydrate when compared to those in the lab (10). German cockroaches in the field will also convergently adapt their dietary preferences to the local environment (11).

Diet can play a significant role in infection and immunity across a range of animals, including insects. For example, in fleas, the specific blood meal source can influence both gut colonization and regurgitative transmission of the human pathogen *Yersinia pestis* (12). In the oriental cockroach, infection with the entomopathogenic bacterium *Pseudomonas entomophila* leads to dietary shifts favoring protein consumption (13). The links between diet and infection are particularly relevant to the role of the German cockroach as a vector for several reasons. German cockroaches harbor a highly diverse bacterial gut microbiota (14), which can be influenced by diet. Diets low in protein have been found to promote bacterial diversity (15), while high-fat diets appear to reduce diversity (16). In general, strong clustering in the composition of the gut microbiota by dietary regimen has been observed in multiple studies (15, 16). In turn, the cockroach gut microbiota can confer colonization resistance against ingested enterobacteria such as *Escherichia coli* and *S.* Typhimurium through mechanisms, including the priming of intestinal innate immunity (7, 17).

There are significant gaps in our understanding of the factors that influence *S.* Typhimurium infection of cockroaches. Given the high variability of diets available to cockroaches across different environments, if diet has significant effects on the ability of cockroaches to become colonized by and disseminate pathogens, this could contribute to differential vector competence among populations. Therefore, it was critical to investigate this hypothesis. To do so, we maintained cockroaches on three defined formulated diets with distinct macronutrient profiles for 10 days. Food consumption was monitored during this period, and the gut microbiome was profiled before we orally infected the cockroaches with *S.* Typhimurium to quantify pathogen loads in the gut and excreta as indicators of vector competence.

## MATERIALS AND METHODS

### Cockroach rearing

The American Cyanamid Orlando laboratory strain of *Blattella germanica* was utilized in all experiments described herein. Cockroach colonies were maintained in plastic arenas at a temperature of 25 ± 1°C with 40%–45% relative humidity on a 12 hour photoperiod. Egg cartons were provided as harborages, and tap water was given *ad libitum*. Prior to experimental changes in diet, cockroaches were maintained on a diet of dog chow (Purina, St. Louis, MO, USA).

### Formulated diets

Three formulated dry diets high in either carbohydrate, protein, or fat were used in this study (Research Diets, Inc., New Brunswick, NJ, USA). The specific macronutrient profiles of each diet are given in Table 1. Corn starch, maltodextrin, and sucrose served as carbohydrate sources. Casein and L-cysteine served as protein sources. Soybean oil and lard served as fat sources. The high-fat diet in this study contained a high amount of sucrose, as it was intended to be high in both fat and sugar content. Each diet also included a mineral mix, dicalcium phosphate, calcium carbonate, potassium citrate, a vitamin mix, and choline bitartrate. These diets were selected to represent variable macronutrient balances using a fixed set of ingredients, not to represent any specific natural cockroach diets, which are largely unknown.

**TABLE 1:** Formulated diets used in this study

Diet	Macronutrients (kcal%)			kcal/g
	Carbohydrate	Protein	Fat	
High carbohydrate	70	20	10	3.85
High protein	50	40	10	4.73
High fat	35	20	45	3.85

### Consumption of formulated diets

Groups of adult male or non-gravid female cockroaches (*N* = 16 of each) were removed from a colony and separated by sex into experimental arenas where they were provided harborage, water, and a large pellet of one of the three formulated diets as a sole food source (no-choice assay). Thus, six separate arenas were prepared. The cockroaches were allowed to consume the diets for 10 days. The mass of each food pellet was recorded before distribution to the cockroaches and at the end of the 10-day period to quantify loss of mass. To determine consumption, loss of mass was normalized for any water loss to the environment using mass recordings from pellets that were kept in arenas without cockroaches for 10 days. The experiment was replicated independently four times. The purpose of this experiment was to ensure that any observed effects of diet in downstream experiments were not confounded by differences in diet palatability. The experiment was not designed with the intent to query or make conclusions about the dietary preferences of cockroaches.

### Microbiome analyses

Whole male and female cockroaches were collected after 10 days on each formulated diet for microbiome analyses. Several prior studies have investigated the specific effects of diet on the microbiome of *B. germanica* (15, 16). Our analyses here were not intended to replicate or refute such findings, but rather to confirm that the diets used were causing some shift in the microbiome, as expected, before proceeding to testing the effects on *Salmonella* infection.

The cockroaches were surface sterilized by successive washes in 10% bleach, 70% ethanol, and water to eliminate external bacteria, then homogenized in sterile phosphate-buffered saline (PBS). Subsequent steps closely followed our previously published protocol for analyses of the microbiome of the brown-banded cockroach (*Supella longipalpa*) (18). In brief, DNA was isolated from cockroach homogenates using a DNeasy blood and tissue kit (Qiagen, Germantown, MD, USA) according to the manufacturer’s protocol. DNA concentration was then measured using a Qubit fluorometer (ThermoFisher Scientific, Waltham, MA, USA). Primers targeting the V4 hypervariable region of the bacterial 16S rRNA gene (515/806) were used for PCR with HotStarTaq Plus Master Mix (Qiagen). Denaturation, annealing, and extension temperatures of 95°C, 53°C, and 72°C, respectively, were used. PCR products then underwent electrophoresis on 2% agarose gel to verify successful amplification. Samples were multiplexed using unique dual indices and pooled together at equal concentrations before being purified using calibrated Ampure XP beads (Beckman Coulter, Brea, CA, USA). Pooled and purified PCR products were used as input for Illumina TruSeq DNA library preparation. Paired-end sequencing (2 × 300) was performed on an Illumina MiSeq instrument (Illumina, San Diego, CA, USA).

16S rRNA sequences were processed using the Qiime2 pipeline (Amplicon version 2024.2, qiime2.org). Raw sequencing data were processed to remove primers and low-quality reads (Phred quality score <25). High-quality reads were considered for denoising, merging, and chimera removal and to generate unique amplicon sequence variants (ASVs) using the Dada2 plugin within Qiime2 (19). Taxonomic assignments of bacterial ASVs were based on reference sequences (clustered at 99% sequence identity) from the Silva 132 reference database (Release 128). A blank control underwent DNA extraction and library preparation and was sequenced to identify any problematic contaminants before downstream analysis. The data analysis presented here was performed at the genus level for consistency with other studies of the German cockroach. To normalize the data, bacterial genera not present in at least three samples were omitted, and the genus table produced was converted to relative proportions using total reads per sample. Microbial community analyses were performed without reads belonging to the extraintestinal symbiont *Blattabacterium* in order to consider only effects on the gut microbiome. Columns of reads assigned to these taxa were subtracted from tables before relative abundance transformation, completely removing them from the analyses while leaving the gut bacterial community intact.

### Administration of *Salmonella* to cockroaches

Cockroaches were orally infected with *S.* Typhimurium as described in our previous work (4, 5, 7). After a 10-day period on different formulated diets, cockroaches were starved of food and water for 3 days prior to infection with *S.* Typhimurium, which promotes consistent oral uptake of the bacteria in liquid media (4). Strain 14028s of *S.* Typhimurium encoding kanamycin resistance was used for all infections in this study. Cultures were grown at 37°C in liquid LB medium containing 100 µg/mL of kanamycin overnight on a shaker under aerobic conditions. Following the growth of *S.* Typhimurium overnight, the culture was diluted to OD600 = 1. This concentration of bacteria has been shown to result in uptake of an average dose of 3.58 × 10^6^ colony-forming units (CFU) per cockroach (4). Blue food dye was added to the cultures prior to providing them to cockroaches to enable non-invasive visual tracking of bacterial consumption. Immediately after the 3-day starvation period, groups of cockroaches were provided *S.* Typhimurium culture in a shallow Petri dish as their only food source for 30 minutes. Following the 30 minute period, the bacterial culture was removed, and the cockroaches were examined for signs of blue dye in the gut to confirm that they consumed the culture. Those that showed no sign of consuming the bacteria were disposed of and excluded from further procedures. Infected cockroaches were maintained for 3 days under the conditions described for colony rearing.

### Determination of internal *Salmonella* loads

Individual cockroaches were collected 3 days post-infection for measurement of internal bacterial loads. Three days post-infection is an important indicator timepoint for susceptibility to *S.* Typhimurium infection in cockroaches. By this point, post-bottleneck bacterial replication has occurred in the cockroach gut (4), and early antimicrobial peptide responses (20) and microbiota-mediated colonization resistance mechanisms (7) have been overcome. The cockroaches were processed to eliminate bacteria present on the cuticle surface by washing them sequentially in 10% bleach, 70% ethanol, and water. Next, each cockroach was placed individually into a 2 mL tube containing 500 µL of sterile PBS and homogenized for 30 seconds using a handheld electronic tissue homogenizer. Homogenates were serially diluted and plated on LB agar containing 100 µg/mL of kanamycin for selective enumeration of *S.* Typhimurium. Plates were incubated overnight at 37°C, after which the number of *S.* Typhimurium CFUs on each plate was counted. Infection prevalence was expressed as the proportion of insects with detectable CFUs, and bacterial loads were calculated as CFU/cockroach based on the CFU counts, the plating factor, and the dilution factor. The theoretical limit of detection of the assay was 500 CFU/cockroach. Samples in which more than 300 colonies were present were considered too numerous to count (TNTC), and 300 was conservatively used as the CFU number for calculating the bacterial load. Four independent replicates were performed for each sex (*N* = 96 males and 114 females total). Uninfected cockroaches served as negative controls and showed no bacterial growth on LB plates with kanamycin.

### Determination of *Salmonella* loads in excreta

Bacterial loads in the excreta of cockroaches during the first 24 hours post-infection were also examined. Immediately following oral infection, cockroaches were gently gripped by the legs using forceps and transferred to individual wells of 12-well tissue culture plates lined with filter paper. Although effort was made to avoid grasping the body of the cockroaches to prevent regurgitation during transfer, any cockroaches that visibly regurgitated were excluded from further analysis. The cockroaches remained in the 12-well plates for 24 hours to allow collection of excreta on the filter paper lining the wells. Excreta were readily visible on the filter paper due to the blue dye added to the bacterial culture. Each filter paper containing visible excreta was folded and placed in a 2 mL tube containing 500 µL of sterile PBS. The filter papers were then incubated at room temperature for 45 minutes with periodic vortex mixing to extract any *S.* Typhimurium present on the filter paper into the PBS. Following the incubation period, the PBS solution was serially diluted and plated on LB agar containing 100 µg/mL of kanamycin for selective enumeration of *S.* Typhimurium. Plates were incubated overnight at 37°C, after which the number of *S.* Typhimurium CFUs on each plate was counted. The prevalence of *S.* Typhimurium excretion was expressed as the proportion of insects with detectable CFUs in the excreta, and bacterial loads were calculated as CFU/cockroach based on the CFU counts, the plating factor, and the dilution factor. The theoretical limit of detection of the assay was 50 CFU/cockroach. Samples in which more than 300 colonies were present were considered TNTC, and 300 was conservatively used as the CFU number for calculating the bacterial load. Two independent replicates were performed for each sex (*N* = 79 males and 76 females total). Excreta from uninfected cockroaches served as a negative control and showed no bacterial growth on LB plates with kanamycin.

### Statistical analyses

All statistical analyses were performed in R (R Core Team, 2023). Data from male and female cockroaches were analyzed separately. Pairwise Fisher’s exact tests were used to determine whether the prevalence of *S.* Typhimurium in cockroaches and their excreta varied significantly following consumption of different formulated diets. *P* values were adjusted for multiple comparisons using Holm’s method. Analysis of variance (ANOVA) was performed on log-transformed CFU values to test for significant differences in the internal bacterial loads and bacteria excreted, with diet as the independent variable. ANOVA was also performed on log-transformed diet consumption data to determine whether there were significant differences in the amount of each diet that cockroaches consumed. Log transformations were necessary in both cases due to deviations from normality among the data. For microbiome data, including alpha diversity, beta diversity, and permutational multivariate analyses of variance (PERMANOVA), multiple R packages such as vegan (21), ape (22), and labdsv (23) were used. The Shapiro–Wilk normality test (shapiro.test function) was used to assess normality, and the proper statistical tests, one-way ANOVA or Kruskal–Wallis test, were used to determine statistical significance among multiple groups. Post hoc Tukey’s HSD or Dunn’s tests with Bonferroni correction were performed to evaluate significant differences among females across diets and separately among males across the same treatments. *P* values <0.05 were considered statistically significant. All figures were generated using the R package ggplot2 (24).

## RESULTS

### Consumption of formulated diets by cockroaches

**Fig 1:**
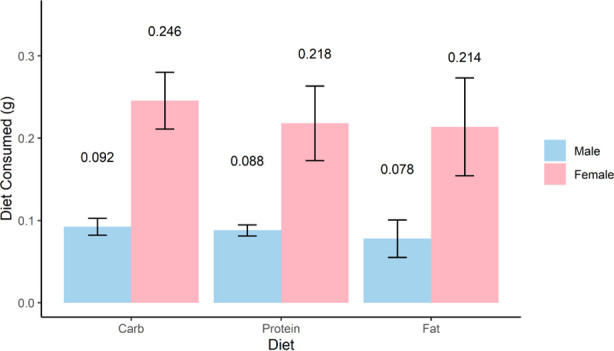
Consumption of formulated diets by uninfected male and female cockroaches. Equal groups of adult male (blue) or female (pink) cockroaches (*N* = 16 each) were provided a dry formulated diet high in carbohydrate, protein, or fat. Consumption of each diet was determined by weight in grams after a 10-day period. Four independent trials were conducted for each group. Bars show the mean and standard error of consumption.

On average, female cockroaches consumed more of each diet than male cockroaches, but no significant differences in consumption of the different diets were observed within either sex in no-choice assays (one-way ANOVA, *P* = 0.29 for males and *P* = 0.567 for females) (Fig. 1). Groups of female cockroaches on high-carbohydrate, high-protein, and high-fat diets for 10 days consumed averages of 0.246, 0.218, and 0.214 g, respectively. Groups of male cockroaches on high-carbohydrate, high-protein, and high-fat diets for 10 days consumed averages of 0.092, 0.088, and 0.078 g, respectively.

### Effects of different formulated diets on the cockroach gut microbiome

**Fig 2:**
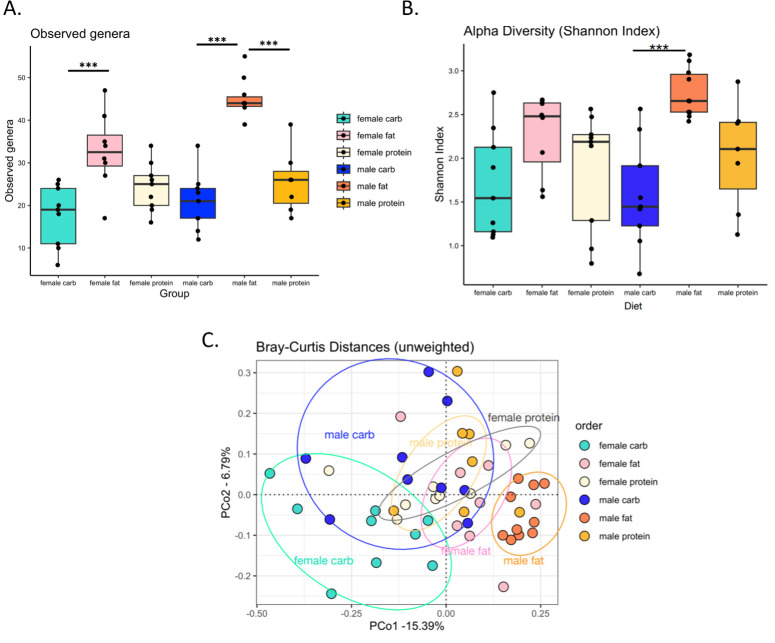
Effects of formulated diets on the gut microbiome of cockroaches. Groups of adult male and female cockroaches were separately maintained on different formulated diets for 10 days then collected for gut microbiome analysis via 16S rRNA amplicon sequencing. (**A**) Differences in observed genera count between cockroaches on different diets. (**B**) Differences in Shannon alpha diversity index between cockroaches on different diets. (**C**) Clustering of cockroaches on different diets based on unweighted Bray–Curtis principal coordinate analysis. Each data point represents an individual cockroach. Observed genera data were analyzed by one-way ANOVA, and Shannon index data were analyzed by Kruskal–Wallis test. Post hoc tests (Tukey’s HSD and Dunn’s test with Bonferroni correction) were used to assess significant differences among cockroaches on different diets separately for each sex. Bray–Curtis distances were analyzed by PERMANOVA. *P* values <0.05 were considered statistically significant as indicated by asterisks in the figures.

There were several differences evident among the gut microbiomes of cockroaches maintained on different formulated diets for 10 days (Fig. 2), which was expected, given previously uncovered impacts of diet on the microbiome of *B. germanica* (15, 16). The high-fat diet significantly increased the number of observed bacterial genera in males relative to both the high-carbohydrate and high-protein diets (one-way ANOVA, *P* < 0.001), as well as in females relative to both diets, although only the difference between the high-fat and high-carbohydrate diets was significant in the latter sex (one-way ANOVA, *P* < 0.001) (Fig. 2A). Similarly, the high-fat diet also significantly increased Shannon alpha diversity in males relative to the high-carbohydrate diet (one-way ANOVA, *P* < 0.001) (Fig. 2B). Unweighted Bray–Curtis principal coordinate analysis further revealed significant clustering by diet (PERMANOVA, *R*^2^ = 0.27, *P* value = 0.001), particularly in males on the high-fat diet and females on the high-carbohydrate diet (Fig. 2C).

### Effects of different formulated diets on *Salmonella* infection and excretion

**Fig 3:**
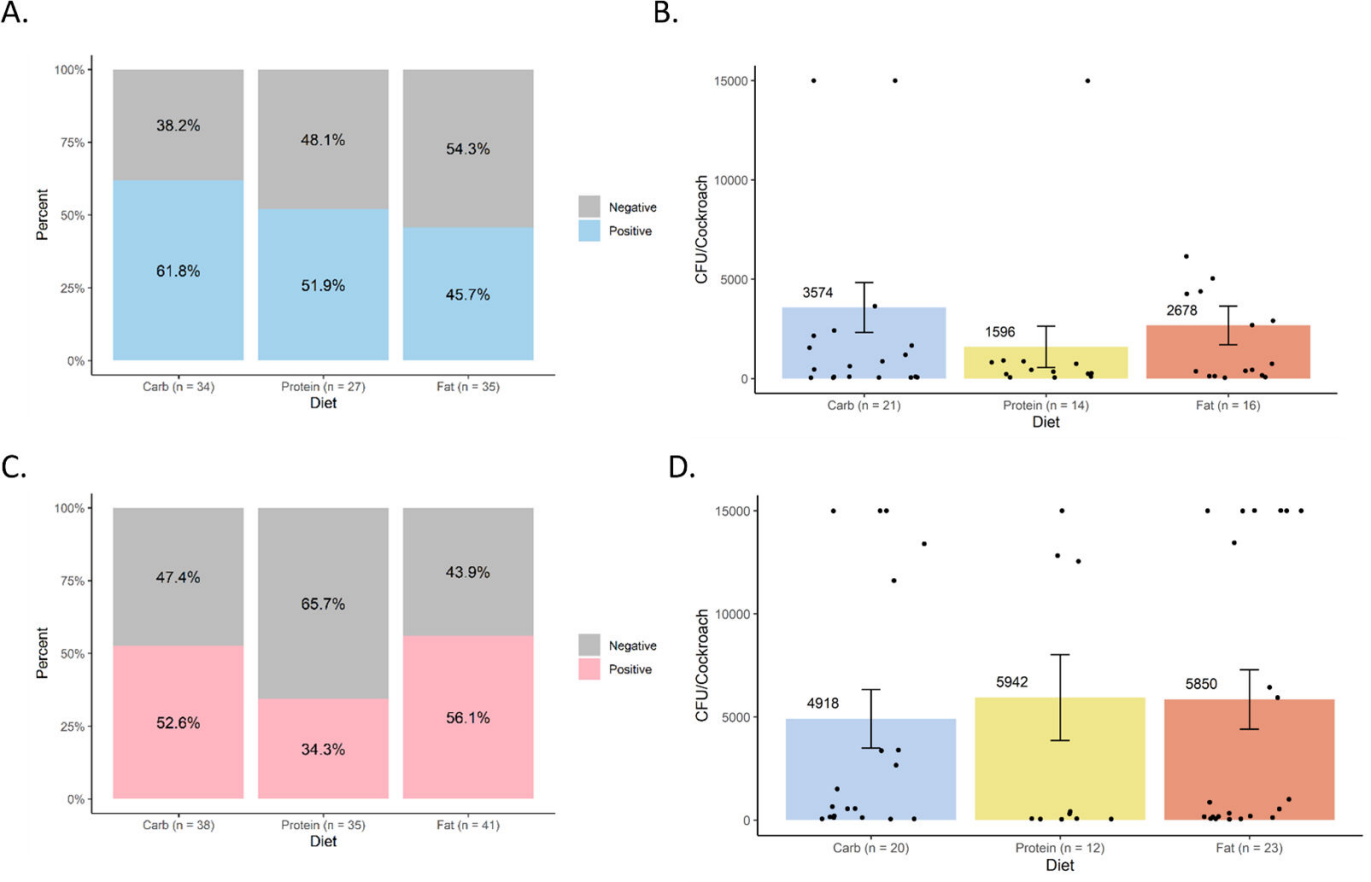
Effects of diet on *Salmonella* infection of cockroaches. Internal *S*. Typhimurium was detected and quantified by selective plating 3 days post-infection in cockroaches that were maintained on different formulated diets (high carbohydrate, high protein, and high fat). (**A**) Infection prevalence in males. (**B**) *S*. Typhimurium load in positive males. (**C**) Infection prevalence in females. (**D**) *S*. Typhimurium load in positive females. Bar graphs in panels B and D display mean bacterial loads (CFU/cockroach) ± standard error. Data points represent individual cockroaches sampled from four independent biological replicates. No statistically significant differences were detected.

Overall, we detected no major nor statistically significant effects of diet on internal or excreted *S*. Typhimurium levels in either cockroach sex. Internal *S*. Typhimurium prevalence and load were assessed 3 days post-infection (Fig. 3). In male cockroaches on the high-carbohydrate, high-protein, and high-fat diets, infection prevalence was 61.8% (*N* = 34), 51.9% (*N* = 27), and 45.7% (*N* = 35), respectively (Fig. 3A). Although the prevalence of infection was highest in male cockroaches on the high-carbohydrate diet and lowest in those on the high-fat diet, pairwise Fisher’s exact tests found that these differences were not statistically significant (*P* > 0.5 for all comparisons). Average internal bacterial loads in infected males (Fig. 3B) were highest on the high-carbohydrate diet (*N* = 21, 3574 CFU/cockroach) and lowest on the high-protein diet (*N* = 14, 1596 CFU/cockroach), mirroring the trend in infection prevalence. However, differences in bacterial load were also not statistically significant (*P* = 0.854). Distinct trends were observed in female cockroaches. For female cockroaches on the high-carbohydrate, high-protein, and high-fat diets, infection prevalence was 52.6% (*N* = 38), 34.3% (*N* = 35), and 56.1% (*N* = 41), respectively (Fig. 3C), but none of the differences between groups were statistically significant (*P* > 0.2 for all comparisons). Average internal bacterial loads in infected female cockroaches did not mirror the prevalence pattern as they did in males (Fig. 3D). Females on the high-protein (*N* = 12; 5,492 CFU/cockroach) and high-fat (*N* = 23; 5,850 CFU/cockroach) diets had higher bacterial loads than those on the high-carbohydrate diet (*N* = 20; 4,918 CFU/cockroach). Again, this trend was not statistically significant (*P* = 0.881).

**Fig 4:**
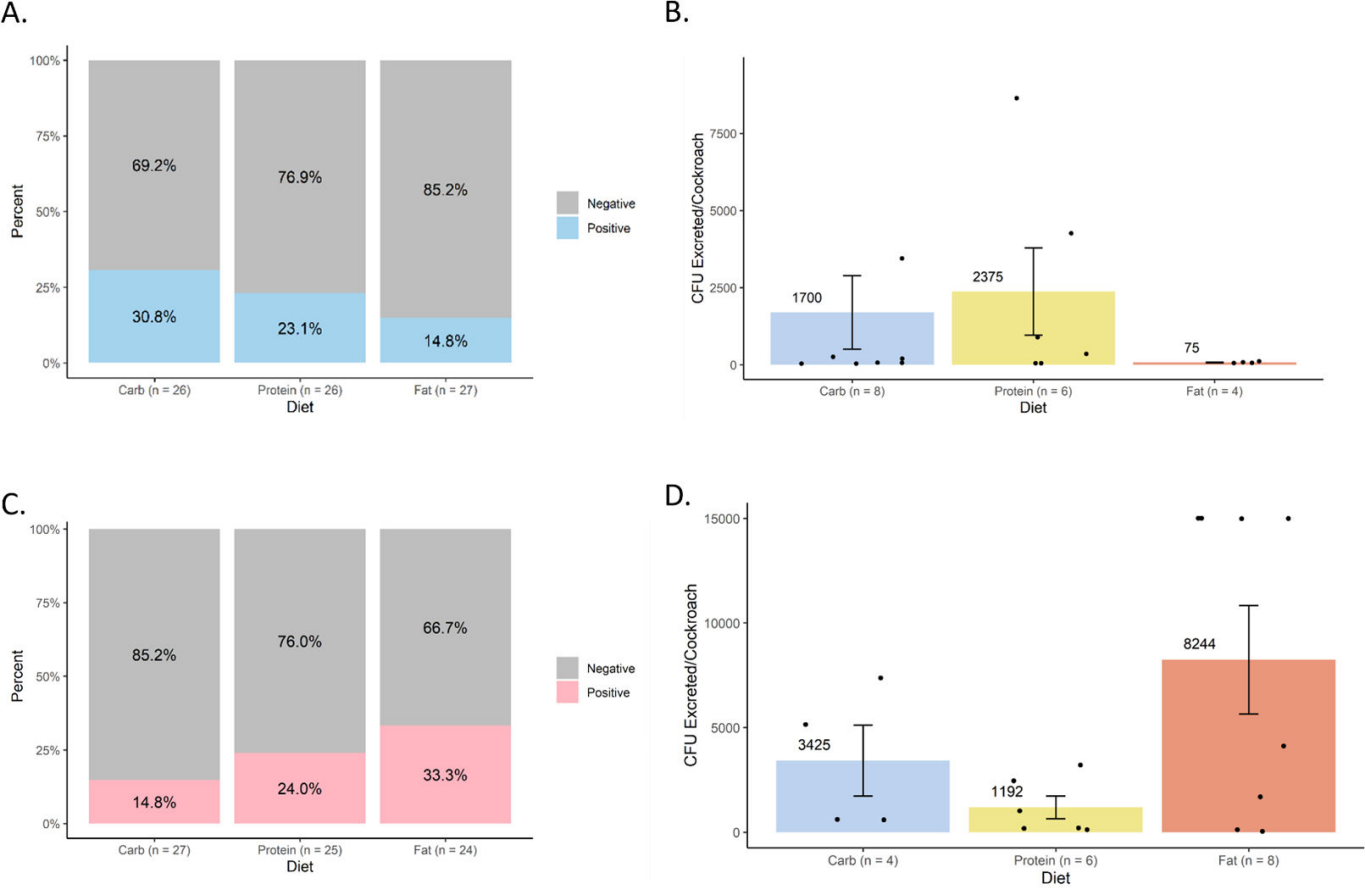
Effects of diet on *Salmonella* excretion by cockroaches. Excreted *S*. Typhimurium was detected and quantified by selective plating for 24 hours post-infection in cockroaches that were maintained on different formulated diets (high carbohydrate, high protein, and high fat). (**A**) Excretion prevalence in males. (**B**) *S*. Typhimurium load in positive excreta of males. (**C**) Excretion prevalence in females. (**D**) *S*. Typhimurium load in positive excreta of females. Bar graphs in panels B and D display mean bacterial loads (CFU/cockroach) ± standard error. Data points represent excreta of individual cockroaches sampled from two independent biological replicates. No statistically significant differences were detected.

The prevalence and load of *S*. Typhimurium within cockroach excreta were assessed 24 hours after infection (Fig. 4). *S*. Typhimurium was present in the excreta of 30.8% of male cockroaches on the high-carbohydrate diet (*N* = 26), 23.1% of male cockroaches on the high-protein diet (*N* = 26), and 14.8% of male cockroaches on the high-fat diet (*N* = 27) (Fig. 4A), but no pairwise comparisons by Fisher’s exact test were significantly different (*P* > 0.6 for all comparisons). The average *S*. Typhimurium loads within positive male excreta were 2,375 CFU/cockroach on the high-protein diet group (*N* = 6), 1,700 CFU/cockroach on the high-carbohydrate diet group (*N* = 8), and 75 CFU/cockroach on the high-fat diet group (*N* = 4) (Fig. 4B), which were not significantly different (*P* = 0.854). As with internal infection, patterns among the prevalence and load of *S*. Typhimurium in female cockroach excreta were distinct from those in male cockroach excreta. Among female cockroaches on the high-carbohydrate, high-protein, and high-fat diets, *S*. Typhimurium was present in the excreta of 14.8% (*N* = 27), 24.0% (*N* = 25), and 33.3% (*N* = 24) of individuals, respectively (Fig. 4C) (*P* > 0.5 for all comparisons). The average bacterial loads within positive female cockroach excreta (Fig. 4D) were highest in cockroaches on the high-fat diet (*N* = 8; 8,244 CFU/cockroach), intermediate those on the high-carbohydrate diet (*N* = 4; 3,425 CFU/cockroach), and lowest in those on the high-protein diet (*N* = 6; 1,192 CFU excreted/cockroach), which were not significantly different (*P* = 0.351).

## DISCUSSION

The results of our dietary consumption and microbiome profiling experiments were consistent with other studies in several ways. We saw that females consistently consumed more resources than males (Fig. 1) (25) and that diet has clear effects on gut microbiome diversity and composition (Fig. 2) (15, 16). On the other hand, our specific observation that the high-fat diet increased microbiome diversity contrasted with the recent findings of Zhu et al., which showed the opposite trend (16). The exact macronutrient profiles and fat sources used (lard and soybean oil vs sesame oil) differed between the two studies, which could have contributed to this disparity.

Despite dietary differences having significant effects on gut microbiota composition and diversity, which indicates that the different diets altered the microenvironment in the gut, we observed no significant impact of diet on *S.* Typhimurium infection or excretion (Fig. 3 and 4). Both infection prevalence and quantitative bacterial loads were considered in males and females, and no statistical significance nor consistent patterns emerged. Notably, bacterial loads at 3 days post-infection were lower than those reported in a 2022 study of the same cockroach strain (4) but strongly aligned with those reported in a more recent 2024 study of the same cockroach strain (7). We attribute these differences to microbiome variation over time and across facilities, as has also been observed to affect susceptibility to *S.* Typhimurium in genetically identical mice from different commercial vendors (7, 26). However, in line with our previous work, internal and excreted *S.* Typhimurium loads did not appear to correlate within treatment groups, which further confirms the decoupling of the gut colonization and excretion processes (6).

Together, our data suggest that diet, particularly macronutrient profile, is not a major driver of vector competence for *S.* Typhimurium, in contrast to the strongly apparent contributions of hologenomic variation between cockroach populations and microbiota-mediated resistance mechanisms unrelated to diet (6, 7). Using the same experimental design used here, the latter variables have been found to drive consistent, exponential, and statistically significant differences in susceptibility to *S.* Typhimurium infection and excretion in cockroaches (6, 7). Changes of such magnitude are particularly biologically meaningful for transmission potential, while changes on the order of only several hundred to several thousand bacteria are less meaningful, given the infectious dose of *S.* Typhimurium to humans is generally reported to be much higher than this. Dietary differences in line with those we tested may not sufficiently alter the balance of specific commensal bacterial taxa that drive susceptibility to *S.* Typhimurium infection (7) nor the metabolic microenvironment needed for *S.* Typhimurium growth in the cockroach gut. Similar to our results, in the oriental cockroach, increased protein consumption following entomopathogenic bacterial infection did not offer any protection (13).

Our study had several limitations that should be considered. In excretion assays, the low number of positive samples and high variability in bacterial loads may have precluded statistical significance when analyzing mean CFUs in the excreta. For example, while not statistically significant, females on the high-fat diet had both a higher prevalence of positive excreta and higher *S.* Typhimurium loads in the excreta than those on the high-carbohydrate and high-fat diets (Fig. 4C and D). Nonetheless, we have detected statistically significant differences in *S.* Typhimurium excretion in other studies with similar sample sizes (4, 6). It is important to consider that analysis of excretion prevalence was more robustly powered but yielded the same statistical result as the data on excreted CFU counts, and that differences between excretion means of 1,000–2,000 CFUs are likely not of major biological significance for human infections. In addition, while we consider using synthetic diets with matched ingredients a strength, other aspects of diet besides macronutrient content, such as micronutrients and fiber, or more extremely unbalanced diets, could influence infection and were not investigated here. For example, in American cockroaches, the gut microbiome is resilient to changes in response to highly unbalanced whole-food diets (27) but is significantly affected by purified fibers in synthetic diets (28).

### Conclusions

The results provide evidence that diet, particularly macronutrient profile, does not have a major effect on cockroach vector competence for *S*. Typhimurium despite its effects on the gut microbiome. However, the possibility that other natural diets could influence pathogen infection and transmission dynamics is not ruled out by this study.

## Data Availability

Raw 16S sequencing data are available in the NCBI SRA under BioProject PRJNA1242374 (https://www.ncbi.nlm.nih.gov/sra/?term=PRJNA1242374).
